# Quality of the Ceramic and Ni-Cr Alloy Joint after Al_2_O_3_ Abrasive Blasting

**DOI:** 10.3390/ma16103800

**Published:** 2023-05-17

**Authors:** Leszek Klimek, Emilia Wołowiec-Korecka, Weronika Czepułkowska-Pawlak, Zofia Kula

**Affiliations:** 1 Institute of Materials Science and Engineering, Faculty of Mechanical Engineering, Lodz University of Technology, B. Stefanowskiego 1/15, 90-537 Lodz, Poland; 2 Department of Dental Technology, Medical University of Lodz, Pomorska 251, 92-213 Lodz, Poland

**Keywords:** abrasive blasting, metal–ceramic bond strength, shear strength, Ni-Cr alloy, thermocycles

## Abstract

The purpose of this in vitro study was to determine the effect of airborne-particle abrasion process parameters on the strength of the Ni-Cr alloy–ceramic bond. One hundred and forty-four Ni-Cr disks were airborne-particle abraded with 50, 110 and 250 µm Al_2_O_3_ at a pressure of 400 and 600 kPa. After treatment, the specimens were bonded to dental ceramics by firing. The strength of the metal–ceramic bond was determined using the shear strength test. The results were analyzed with three-way analysis of variance (ANOVA) and the Tukey honest significant difference (HSD) test (α = 0.05). The examination also considered the thermal loads (5000 cycles, 5–55 °C) to which the metal–ceramic joint is subjected during exploitation. There is a close correlation between the strength of the Ni-Cr alloy–dental ceramic joint and the alloy roughness parameters after abrasive blasting: Rpk (reduced peak height), Rsm (the mean spacing of irregularities), Rsk (skewness of the profile) and RPc (peak density). The highest strength of the Ni-Cr alloy surface bonding with dental ceramics under operating conditions is provided by abrasive blasting under 600 kPa pressure with 110 µm Al_2_O_3_ particles (*p* < 0.05). Both the abrasive blasting pressure and the particle size of the Al_2_O_3_ abrasive significantly affect the joint’s strength (*p* < 0.05). The most optimal blasting parameters are 600 kPa pressure with 110 µm Al_2_O_3_ particles (*p* < 0.05). They allow the highest bond strength between the Ni-Cr alloy and dental ceramics to be achieved.

## 1. Introduction

The bond strength between the metal substrate and dental ceramic in metal–ceramic prosthetic restorations determines their service life during exploitation in the patient’s mouth. Several factors are crucial for the bond strength, including chemical bonds between the joined materials and stresses present in the bond, caused by the difference in coefficients of material thermal expansions (CTE) [1,2,3,4,5,6]. However, it is the roughness of the surface that most significantly influences the durability of the joint. It provides points for the mechanical anchoring of the ceramic during fusion [1,7]. The proper roughness is obtained by abrasive blasting during the preparation of the metal surface for bonding to the ceramic. The airborne-particle abrasion process is a clinically acceptable method of Ni-Cr surface preparation before dental ceramic firing.

Numerous studies have indicated that parameters used in abrasive blasting differently affect the surface of metals used in prosthetics and the strength of the metal–ceramic joint [7,8,9,10,11,12,13,14]. According to Gołębiowski and Pietnicki, treatment with 110 μm aluminum oxide under a pressure of 400 kPa provides the best bond strength values for titanium and cobalt–chromium alloys [8,9], which contradicts the hypothesis that the higher the abrasive blasting parameters, the better the bond strength [13].

The good mechanical and technological properties of nickel–chromium alloys are the reason they have long been used in dental prosthetics. The main benefits are the long-term and failure-free use of prosthetic restoration for patients and comfortable work with the material for the dental technician who makes the restoration. However, it must be noted that there has been a tendency to refrain from using alloys containing nickel due to the risk of allergies in patients [15,16]. However, when we consider the favorable properties of these alloys, it seems that they can be used for prosthetic restorations on the condition of applying surface treatments that improve biocompatibility and reduce the risk of allergy. Such research is conducted with coatings (oxide, carbon, nitride, carbide, etc.) used to cover alloys of various metals [17,18]. The conducted research shows that applying such coatings reduces the harmful effects of nickel, so these alloys can be confidently used after appropriate surface abrasive blasting [19,20,21,22]. The laid procedures will also enable additional properties to be obtained, such as a reduction in bacterial biofilm formation and an improvement in abrasion resistance or fretting wear [22,23].

Considering the possibility of the further use of nickel–chromium alloys in prosthodontics, it seems essential to investigate the joint strength of these alloys with veneering ceramics. There are many studies on the joint of veneering ceramics with cobalt, titanium, or zirconium oxide alloys subjected to various abrasive blasting procedures. However, there are no literature reports on the analysis of the effect of the abrasive blasting of these alloys on their joints with ceramics. 

This study aimed to analyze the influence of different parameters of abrasive blasting on the strength of a metal–ceramic joint and to investigate the effect of thermal loads on its durability.

## 2. Materials and Methods

One hundred and forty-four Heraenium^®^ NA nickel–chromium alloy specimens (Heraeus Kulzer, Hanau, Germany) were formed as cylinders with a diameter of 8 mm and a height of 15 mm. The chemical composition is presented in Table 1. The X-ray fluorescent analysis method used an SRS300 spectrometer (SIEMENS, Berlin, Germany) to determine the alloy’s chemical composition. Specimens were subjected to abrasive blasting (Alox 2001, Effegi Brega, Sarmato, Italy) using aluminum trioxide (Al_2_O_3_) for 20 s, with a nozzle inclination at 45° and a distance of 15 mm from the surface of the material. Then, they were divided into six subgroups (n = 24). The groups were distinguished by abrasive blasting parameters, where the abrasive particle size and the processing pressure were the variables (Table 2). After abrasive blasting, all specimens were cleaned in an ultrasonic cleaner (Emmi-55HC-Q, Emag, Poland) in deionized water for eight minutes to remove loose abrasive particles. Then, the surface was dried under compressed air. Then, IPS Classic^®^ dental ceramics (Ivoclar Vivadent, Schaan, Lichtenstein) were fused to such prepared surfaces in the form of two opaque layers and two dentin layers according to the manufacturer’s recommendations (Table 3). 

Each group was divided into two equally significant subgroups. One subgroup in each group (n = 12) was subjected to thermal loads (thermocycles) involving alternate immersion of the specimens in baths of 5 °C and 55 °C. Five thousand cycles of temperature changes were performed. The other subgroups were left intact. Representatives from all groups were then subjected to a shear strength test of the dental metal–ceramic bond (Zwick/Roell Z020, Zwick, Ulm, Germany). Statistical analyses were conducted with the Statistica software (StatSoft, Tulsa, OK, USA). A 3-factor ANOVA and a post hoc Tukey test were conducted (α = 0.05). After the shear strength tests were completed, the specimens were subjected to fractographic tests, which consisted of observing the surfaces of obtained fractures in a scanning electron microscope, JEOL JSM-6610LV (JEOL, Tokyo, Japan). In addition, the spatial distribution of elements was analyzed on the surfaces of the fractures to determine the nature and location of the joint fractures.

## 3. Results

**Figure 1:**
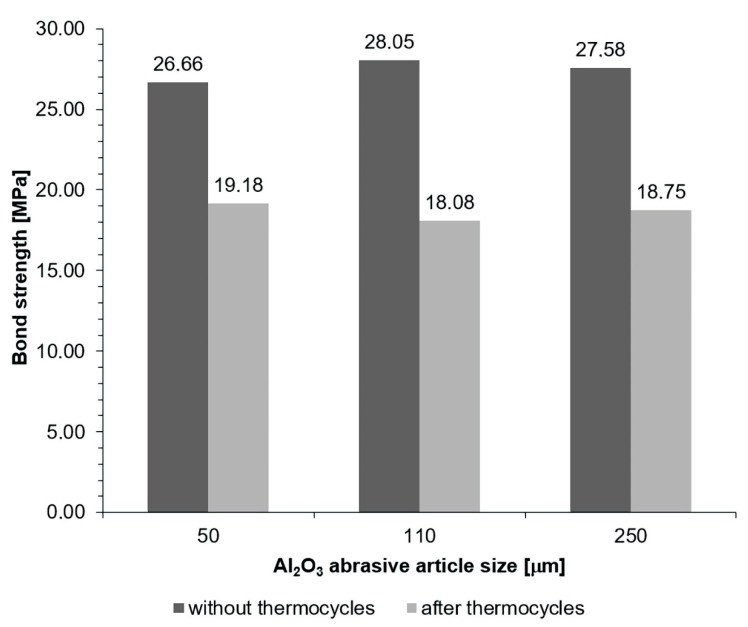
Results of shear strength measurements of the Ni-Cr alloy–dental ceramic joint for a pressure of 400 kPa.

**Figure 2:**
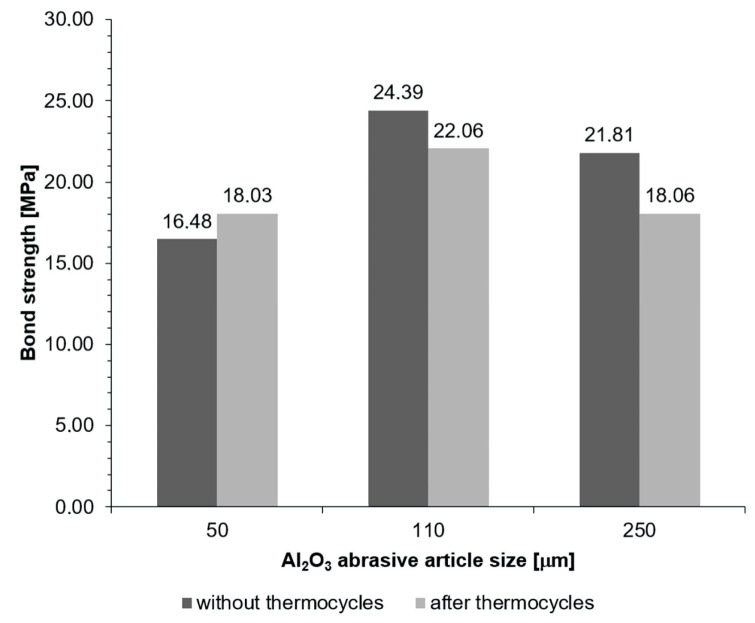
Results of shear strength measurements of the Ni-Cr alloy–dental ceramic joint for a pressure of 600 kPa.

The content of Table 4, as well as Figure 1 and Figure 2, show the results of the shear strength tests of the Ni-Cr alloy–dental ceramic joint. The abrasive treatment with a grain size of 50 µm under a pressure of 600 kPa provided the highest shear strength values for joints not subjected to thermal loads. The bond strength was similar for treatment under a pressure of 400 kPa, irrespective of the grit size. A significant difference in the strength was proved in the group after treatment with a grain size of 50 µm and other grits under a pressure of 600 kPa (*p* < 0.05).

For joints subjected to thermal loads (which is the most important factor regarding a long joint life in the patient’s mouth), the highest shear strength values were observed for specimens blasted with the smallest abrasive grit size (50 µm) under the pressure of 600 kPa. The bond strength was similar for a pressure of 400 kPa, irrespective of the grit size. A significant difference in the strength was observed in the group blasted with a grit size of 110 µm and other sizes under a pressure of 600 kPa.

For joints prepared by abrasive blasting under 400 kPa pressure, the strength of joints without thermocycles (no thermal loads) is higher than after blasting under 600 kPa pressure. However, the strength of joints prepared under 400 kPa pressure and then subjected to thermal loading is significantly reduced (*p* < 0.05), which is not observed for 600 kPa pressure. Moreover, there is a relationship between pressure and abrasive particle size (*p* < 0.001), as well as a relationship between pressure and thermal load (*p* < 0.001) (the size of one parameter may positively or negatively affect the other).

**Figure 3:**
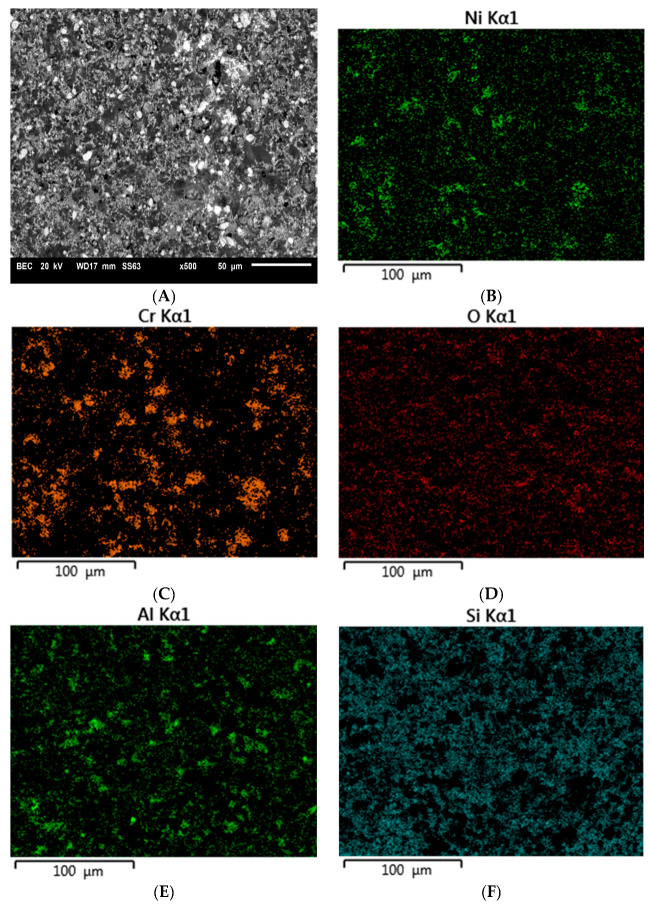
Microscopic image including surface elemental distribution of the fracture of specimen A45 (400 kPa/50 μm): (**A**) general view, (**B**) Ni, (**C**) Cr, (**D**) O, (**E**) Al, (**F**) Si.

**Figure 4:**
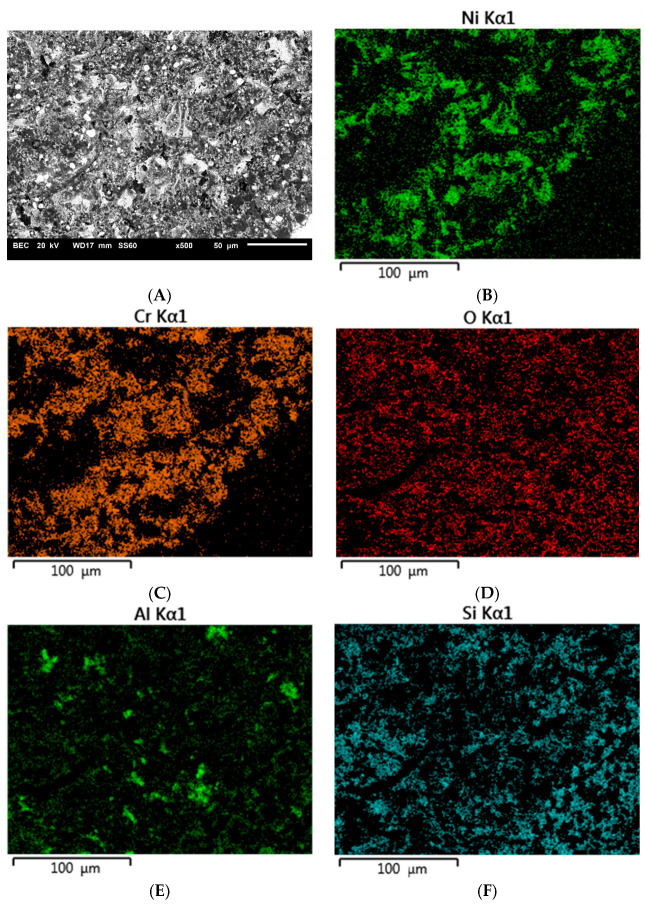
Microscopic image including surface elemental distribution of the fracture of specimen A41 (400 kPa/110 μm): (**A**) general view, (**B**) Ni, (**C**) Cr, (**D**) O, (**E**) Al, (**F**) Si.

**Figure 5:**
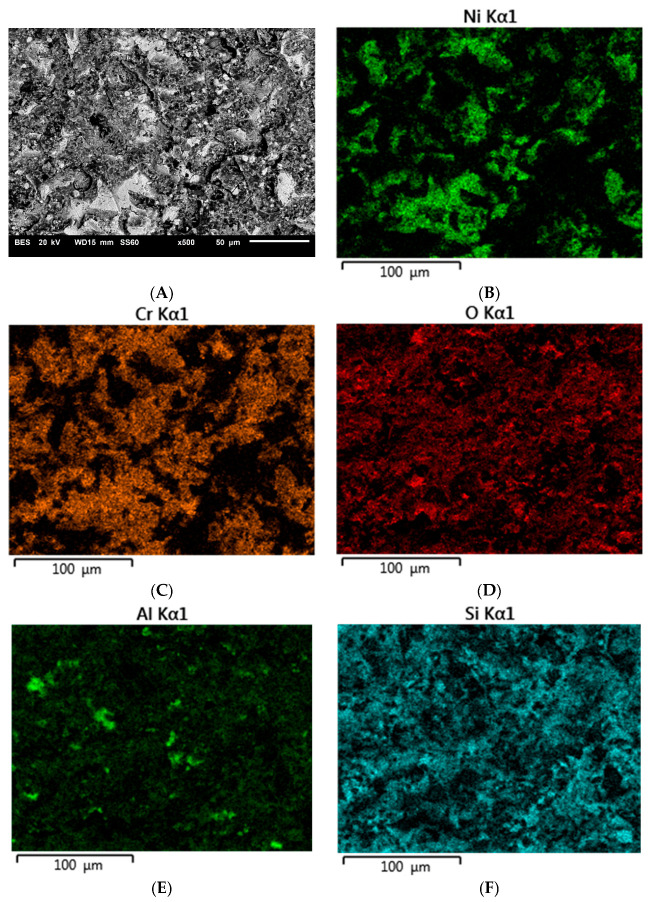
Microscopic image including surface elemental distribution of the fracture of specimen A42 (400 kPa/250 μm): (**A**) general view, (**B**) Ni, (**C**) Cr, (**D**) O, (**E**) Al, (**F**) Si.

Figure 3, Figure 4 and Figure 5 show example images of fracture surfaces, including surface elemental distributions.

The elements contained both in the metal substrate (Ni, Cr) and the ceramics (Si, Al) were observed on the microscopic fracture images. It should be interpreted that during the shear test, the fracture occurred both through the ceramics and the metal, as well as at the interface between the metal substrate and veneering ceramics. Similar observations were made for specimens subjected to thermocyclic testing and investigated by the shear test.

## 4. Discussion

### 4.1. Shear Strength and Its Dependence on Thermal Loads

Investigations of the shear strength of a joint between a dental ceramic and a Ni-Cr alloy indicate that abrasive blasting parameters and thermal loads significantly affect the durability of the joint. A study by Pietnicki et al. [8] on the shear strength of a cobalt–chromium alloy and dental ceramic joint shows that for this material, the best blasting parameters for abrasion with Al_2_O_3_ particles include a pressure of 400 kPa and grit size of 110 µm. The same result was obtained in a study conducted by Gołębiowski et al. [9] on a joint between titanium and dental ceramics. The results of the investigations presented in this paper indicate that a pressure of 600 kPa and grit size of 110 µm provide the best results for the Ni-Cr alloy for its practical applications.

Tests conducted on the shear strength of metal–ceramic joints after subjecting them to thermal loads also confirmed a relationship between the strength and abrasive blasting parameters. For most surfaces, the strength decreased, except after blasting with 50 µm grit size and 600 kPa pressure. However, for this surface, the results obtained without and after thermocycling are not significantly different. The decreased strength of the joint after thermocycles was also observed for the joint between veneering ceramics and titanium [24,25,26,27], gold [25,28,29,30], or cobalt–chromium alloy [27,28,31]. Thermal loading affects the strength of the joint by causing repeated weakening stresses at the metal–ceramic interface [24]. This is due to differences in the coefficients of thermal expansion of the materials used to create the restorations [28]. Repeated cyclic loading might lead to the formation of microcracks at the metal–ceramic interface. Such cracks reduce the actual surface area of the joint, which results in the reduction of its strength. The analysis of microscopic images of cracks in the samples after the shear test confirmed that the cracks occurred primarily at the contact of the metal substrate with the veneering ceramics. The proportion of individual fracture areas varies for specimens blasted with different parameters. Such complex crack propagation does not allow us to unequivocally identify the weakest link in the tested joint. It could be said that this is a favorable situation and all the elements of the system are equally responsible for the strength of the joint.

### 4.2. Influence of Surface Roughness Parameters on Shear Strength

**Figure 6:**
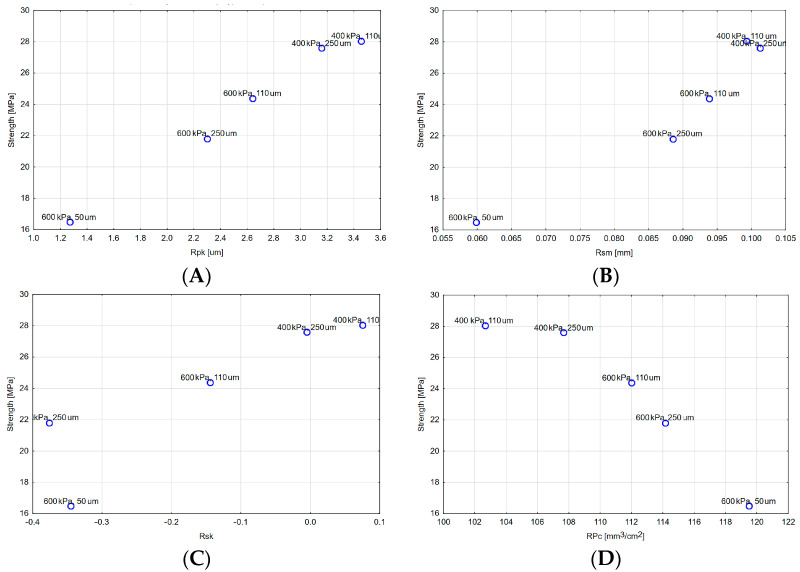
Graph of the strength scatter of the Ni-Cr alloy–ceramic joint in relation to surface roughness parameters: (**A**) Rpk, (**B**) Rsm, (**C**) Rsk, (**D**) RPc.

The obtained results of the strength tests were compared to the surface roughness parameters of the Ni-Cr alloy before fusing the ceramics. They are presented in detail in a work by Czepułkowska et al. [13]. A comparative analysis of the results of the joint strength and alloy surface roughness parameters revealed that with the exception of a specimen blasted under 400 kPa pressure and with a 50 µm grit size, there is a close positive linear correlation between the strength and the surface roughness parameter Rpk, that is, the average height of peaks protruding above the roughness core profile. The value of the Pearson correlation coefficient for this parameter is 0.995. The coefficient of determination is 0.9895, which means that the strength is 99%, explained by the variability of the Rpk parameter. The significance level *p* for the t-statistic is less than 0.05, which means that the correlation coefficient is significantly different from 0. Slightly weaker correlations were observed for the following parameters: Rsm (the mean spacing of irregularities) (0.9259), Rsk (skewness of the profile) (0.7889) and RPc (peak density) (−9.9091). For all parameters, except for RPc, the correlation is positive, which means that increasing values of blasting parameters were accompanied by increasing values of roughness parameters. For the RPc parameter, a negative correlation was observed (Figure 6).

It seems that the effect of these parameters on strength can be explained on the base of their physical properties. Undoubtedly, the reduced peak height (Rpk) influences the anchorage of liquid ceramics in irregularities. The greater this height is, the greater the contact area between the metal substructure and the ceramic will be. An analysis of fractures observed after the shearing test indicates that, in most cases, a fracture occurs at the metal–ceramic interface (adhesive fracture), so increasing this surface area increases the strength of the joint. Moreover, greater heights are identified with the stronger anchorage of the ceramics in metal irregularities. The mean spacing of irregularities (Rsm) conditions the ability of the ceramics to flow into the irregularities. Taking the capillary forces and the wettability into account, it can be concluded that only the right width of the irregularities will enable the ceramics to flow into them. This will not be possible if the grooves are too narrow. It should be expected that the greater the width, the greater the strength. It also seems to be obvious that from a certain value of the groove width, a further increase in its width will have no effect on the strength of the joint or may even reduce it. Thus, we can definitely state that there is a relationship between the strength of the joint and the width of the groove for values obtained during the abrasive blasting procedure of prosthetic components. The skewness of the profile coefficient, considered in the context of mechanical engineering, relates to the retention of lubricant on the component surface. At fusion temperature, the ceramic is liquid. Thus, this coefficient is responsible for the retention of the liquid ceramic in surface irregularities, which has a direct impact on the strength of the joint. The effect of the peak density (RPc) can partly correspond to the influence of the profile height. The more significant the number of peaks, the larger the contact area. Furthermore, in the case of cohesive fracture, a more significant number of peaks indicates a larger cross-sectional area that can be affected by a cohesive fracture, so a more considerable force is required for this fracture to occur.

Abrasive blasting changes the surface condition of the abraded material. A study on the effect of sandblasting on a Ni-Cr alloy revealed that basic roughness parameters, such as Ra and Rz, for procedures with the application of 400 and 600 kPa pressures are almost the same, and their value increases with an increase in the grit size [13]. On the basis of an analysis of graphs demonstrating the strength of the joint depending on the applied parameters, we can conclude that these roughness parameters are too general and should not be used while analyzing the joint between metal and dental ceramics. The same conclusion can be drawn from the study by Gołębiewski et al. [9]. The authors describe roughness with Ra and Rz parameters and conclude that they do not affect joint strength. The Rsm parameter, which relates to the mean spacing of irregularities, may appear to be the most useful roughness parameter. A comparison of the study graphs [13] enables us to conclude that for the grit sizes 110 and 250 µm, different values of the Rsm parameter for both pressures are insignificant. A similar observation was made for the joint strength values. Differences are observed for the smallest grit size, which may be due to different surface properties. Additionally, the Lr coefficient and the V_o_ (oil volume of a surface) parameter can be helpful in analyzing the surface conditions, which are required to create joints in prosthetic restorations. Another surface property, i.e., the surface free energy, reveals significant differences between surfaces after sandblasting at a pressure of 400 and 600 kPa for larger grit sizes. However, it does not translate into strength results. Similarly, no effect of abrasive particles embedded in the surface is observed. In summary, mechanisms occurring at the metal–ceramic interface during the fusion procedure are extremely complex, and most parameters describing the surface condition appear to be inadequate. Only parameters describing the width of the irregularities or the oil volume of the irregularities can influence the final value.

In conclusion, for a nickel–chromium alloy, the very appropriate blasting pressure is important only for the smallest abrasive grit size (50 µm). For the other tested machining parameters, no significant differences in the shear strength of the metal–ceramic interface are observed, which allows any of the sandblasting variables to be applied to create a durable prosthetic restoration. Thermal loading significantly reduces the bond strength.

## 5. Conclusions

Based on the results presented in this study, the following conclusions were drawn:

1. There is a close correlation between the strength of the Ni-Cr alloy–dental ceramic joint and the alloy roughness parameters after abrasive blasting: Rpk, Rsm, Rsk and RPc.
2. The application of abrasive blasting under 600 kPa pressure makes the alloy–ceramic joint durable in the operating conditions under thermal load.
3. The most optimal blasting parameters are 600 kPa pressure with 110 µm Al_2_O_3_ particles (*p <* 0.05). These parameters allow the highest bond strength to be achieved between the Ni-Cr alloy and dental ceramics.
4. Variable thermal load reduces the strength of the bond, irrespective of abrasive blasting parameters.

## Figures and Tables

**Table 1:** Heraenium^®^ NA alloy as research material (chemical composition, wt.%).

Mo	Fe	Ta	Si	Co	Cr	Mn	Nb	Ni
9.21	1.53	0.19	1.54	0.15	24.63	0.42	0.48	residue

**Table 2:** The parameters of abrasive blasting processes.

Al_2_O_3_ Abrasive Particle Size [µm]	Processing Pressure [kPa]	
	400	600
50	A45	A65
110	A41	A61
250	A42	A62

**Table 3:** The parameters of ceramic firing. V1—vacuum start temperature, V2—vacuum end temperature.

Layer No	Temp. (Max) [°C]	Resting Temp. [°C]	Drying Time [min]	Rise Temp. [°C]	Time [min]	V1 Temp. [°C]	V2 Temp. [°C]
Opaque							
I	980	403	6	80	1	550	979
II	970	403	6	80	1	550	969
Dentine							
I	920	403	4	60	1	580	919
II	910	403	4	60	1	580	909

**Table 4:** Shear strength measurements and their impact on the Ni-Cr alloy–dental ceramic joint. Statistically significant differences compared with 50 um/400 kPa are indicated by different letters.

Pressure [kPa]	Al_2_O_3_ Particle Size [µm]	Bond Strength [MPa] (Mean ± SD)		
		No Thermocycles	Thermocycles	Total (Particle Size × Pressure)
400	50	26.66 ± 5.49	19.18 ± 2.55	22.92 ± 5.67
400	110	28.05 ± 3.83	18.08 ± 3.43	23.07 ± 6.21
400	250	27.58 ± 2.99	18.75 ± 3.44	23.17 ± 5.50
600	50	16.48 ± 3.39	18.03 ± 3.98	17.25 ± 3.70
600	110	24.39 ± 4.49	22.06 ± 3.23	23.22 ± 4.01
600	250	21.81 ± 4.53	18.06 ± 2.31	19.93 ± 4.01
Total (Thermocycles)		24.16 ± 5.74	19.03 ± 3.41	21.59 ± 5.36
3-factor ANOVA				
Factor	F	*p*	Partial eta2	Power
Pressure	21.92	0.000	0.142	0.996
Particle size	8.03	0.001	0.108	0.953
Thermocycles	67.90	0.000	0.340	1.000
Pressure × Particle size	7.35	0.001	0.100	0.934
Pressure × Thermocycles	33.81	0.000	0.204	1.000
Particle size × Thermocycles	3.04	0.051	0.044	0.580
Pressure × Particle size × Thermocycles	0.86	0.426	0.013	0.195
